# Single-Leg Balance and Lower Limb Strength: Quantitative Analysis with the Balance Master System

**DOI:** 10.3390/jfmk9040282

**Published:** 2024-12-21

**Authors:** José Manuel Delfa-de-la-Morena, Pedro Pinheiro Paes, Débora Priscila Lima de Oliveira, Frederico Camarotti Júnior, Bruna Daniele Monteiro Lima, Miriam García-González, Juan-José Mijarra-Murillo, Víctor Riquelme-Aguado

**Affiliations:** 1 Department of Physical Therapy, Occupational Therapy, Rehabilitation and Physical Medicine, Health Sciences Faculty, Rey Juan Carlos University, 28922 Madrid, Spain; jose.delfa@urjc.es (J.M.D.-d.-l.-M.); miriam.garciag@urjc.es (M.G.-G.); 2 Cognitive Neuroscience, Pain, and Rehabilitation Research Group (NECODOR), Faculty of Health Sciences, Rey Juan Carlos University, 28922 Madrid, Spain; 3 Department of Physical Education, Federal University of Pernambuco, Recife 50670-901, Brazil; pppaes@ufpe.br (P.P.P.); debora.limaoliveira@ufpe.br (D.P.L.d.O.); frederico.camarottijunior@ufpe.br (F.C.J.); bruna.daniele@ufpe.br (B.D.M.L.); 4 Research and Studies in Health and Performance Group (GEPPHS), Federal University of Pernambuco, Recife 50670-901, Brazil; 5 International Doctoral School, Faculty of Health Sciences, Rey Juan Carlos University, 28922 Madrid, Spain; 6 Department of Basic Health Sciences, Health Sciences Faculty, Rey Juan Carlos University, 28922 Madrid, Spain; victor.riquelme@urjc.es; 7 Research Group on Anatomical, Molecular and Human Development Bases (GAMDES), Rey Juan Carlos University, 28922 Madrid, Spain; 8 Fisioterapia Oreka CB, 45200 Illescas, Spain

**Keywords:** body composition, metabolic equivalent of task, motor skills, postural balance

## Abstract

**Objective**: This study investigates the relationship between lower limb strength and postural stability in single-leg stance using the Balance Master system. **Methods**: The research involved 64 participants divided into sedentary and physically active groups based on metabolic equivalents of task (METs) values, normal weight, overweight, and obese according to body composition. Postural control was evaluated using the Sensory Organization Test. **Results**: The results showed that there were no significant differences in mean and maximum lower limb strength between the groups. Furthermore, postural stability in open and closed eyes conditions did not show significant differences between the groups. However, a significant positive correlation was observed between lower limb strength and stability in single-leg stance with eyes open. **Conclusions**: These findings suggest that lower limb muscle strength is essential for postural stability, especially when vision is available to aid balance. The study highlights the importance of interventions focused on strengthening muscles to improve physical functionality in adults.

## 1. Introduction

Balance is characterized as the ability to maintain the center of body mass (CM) or center of gravity (CG) projected vertically on a support base within the limits of stability [1]. The CM is determined by the average weight of each body segment, which is supported by the area delimited by the lateral edges of the feet in contact with a support surface (BA), thus respecting the limits of body stability [2]. Once these conditions are disturbed, balance is impaired, and the risk of falls increases, becoming a worrying public health issue in the adult and elderly population [3].

Postural balance is essential for daily activities, motor development, and the practice of sports activities. To perform physical and sports activities, maintaining balance and body orientation is necessary [1]. The balance provided by the feet refers to the ability to maintain the center of gravity within the support base. The corrections of the body axis by postural control mechanisms, caused by the dynamics of the living organism itself, give the human body small and constant oscillations when standing, with an important role in the distribution of pressure on the soles of the feet [4].

During single-leg stance (SLS), the muscles of the supporting lower limb have to exert large amounts of force and contribute to postural control in order to maintain a stable posture, which is essential for daily functionality and fall prevention [5]. Unipodal support is mostly considered to be static balance (due to the body’s ability to maintain a certain posture), but in practice, it also involves a dynamic component due to the small regulations of contralateral oscillations [3,6]. Lower limb disorders and body composition changes can decrease lower limb strength or balance ability, making postural stability extremely difficult [7].

Strain et al. [8] demonstrated national and global trends in physical activity in adults; 5.7 million participants from 2000 to 2022 showed that insufficient levels increased from the age of 60 in all regions. This is also related to the growing rates of overweight and obesity. Locality, due to its socio-economic and cultural aspects and inequalities, also plays an important role in adherence to a more active lifestyle.

When it comes to balance, its relationship with muscle strength and body weight is complex and multifactorial, influenced by the individual’s physical activity levels and involved in various other issues such as biomechanics, age, neurology, and cognition [5]. Neuromuscular coordination is fundamental due to the interaction it promotes between the central nervous system and the musculoskeletal system, interfering in the proprioception of joint mechanoreceptors and sensory neurons in the adjustments of activation and recruitment of fibers and muscle groups [6,9].

Confidence and concentration are also important psychological factors for good balance. People who are more confident and concentrated (by nature or through mental training) demonstrate body actions with a better interpretation of sensory information (visual, vestibular, proprioceptive), more security and autonomy, less fear of falls, and a greater preventive and anticipatory motor repertoire [10,11].

Postural control and balance depend on the integration of responses from the visual, vestibular, and proprioceptive systems, which complement each other, i.e., vision, hearing, muscles, and joints [12]. The vestibular system is located in the inner ear. It works as a “sensor” of the position and movements of the body and head in relation to the force of gravity and other external forces that may disturb postural stability, as well as making rapid adjustments at times of sudden movements [13].

The visual system, in turn, through the eyes and central and peripheral vision, provides information on the spatial orientation of the body in a given environment, allowing the central nervous system (CNS) to make the necessary adjustments to adapt to the different types of surfaces and objects that need to be crossed [12]. The proprioceptive system, as a complement to the visual and vestibular systems, generates information through joints and muscles about position and movements, allowing the CNS to perceive the positioning of body limbs without necessarily looking, helping with postural adjustments and preventing falls [12,13].

Human balance, the ability to maintain an upright posture, is unstable because it is subject to various forces, both internal and external, which disturb stability, and in order to compensate for this, it is necessary for the muscular forces, especially of the lower limbs, which form part of the body’s support base and can modulate greater or lesser postural instability, to be able to act efficiently [14].

There are few studies in the literature on the relationship between lower limb strength and (unipodal) balance in adults. The articles in this context are focused on the elderly, the prevention of falls and injuries in this age group, and athletes. This justifies the importance of this study in evaluating the relationship between muscle strength and balance, serving as a basis for future research and interventions in this segment aimed at improving physical functionality in adults. In view of the above, the aim of the study was to evaluate the relationship between lower limb strength and stability in single-leg stance in different (eyes open and eyes closed) conditions, considering variables such as physical activity level and body weight classifications.

## 2. Materials and Methods

### 2.1. Study Design

This study is observational and cross-sectional, with consecutive non-probabilistic inclusion of cases. The study protocol, in accordance with the Declaration of Helsinki regarding research involving human subjects, was approved by the Ethics Committee of Rey Juan Carlos University (number: 300120170241). The trial is registered on ClinicalTrials.gov with the ID: NCT01116856 (http://clinicaltrials.gov/study/NCT01116856, accessed on 5 May 2010).

### 2.2. Participants

The study included 64 male participants aged between 25 and 60 years, with a body mass index (BMI) ranging from 18 to 35 kg/m² and stable body weight (defined as no weight gain or loss of 2 kg in the past three months). Exclusion criteria comprised individuals with serious illnesses, smokers or recent ex-smokers (within the past six months), alcohol consumption, a history of balance disorders, knee or hip replacement surgery, lower limb trauma within the last six months, or arthritis or other severe inflammatory diseases in the lower limbs. An invitation to volunteer for the study was sent via email to 325 individuals who had previously expressed interest in participating in the PRONAF (Nutritional and Physical Activity Program for Obesity Control) study. Of the 131 individuals who responded, 67 met the inclusion criteria. During data collection, four participants withdrew for personal reasons, resulting in a final sample of 63 subjects. All participants were provided with written information outlining the nature and purpose of the study and gave their informed consent prior to the commencement of the research.

### 2.3. Study Variables

#### 2.3.1. Antropometric Variables

Anthropometric variables included weight and height. Weight was measured using a Tanita^® ^ BC-420MA scale (Bio Lógica Tecnología Médica S.L, Barcelona, Spain), and height was measured using a Seca^®^ height rod (range: 80–200 cm; Seca GmbH & Co. KG, Hamburg, Germany). Body mass index (BMI) was calculated from these measurements [15]. Participants were classified as obese if their BMI was ≥30 kg/m², overweight if their BMI was between 25 and 29.9 kg/m², and normal weight if their BMI was <25 kg/m².

#### 2.3.2. Physical Activity Level

Physical activity was assessed using accelerometry with the SenseWear^®^ Armband (SWA) (BodyMedia Inc., Pittsburgh, PA, USA), a multisensory, objective device that is valid and reliable for measuring physical activity [16,17]. The Physical Activity Level (PAL) test [18] was used to classify participants as either sedentary or active for comparative analysis. Participants with a PAL classification of “not very active”, “active”, or “very active” (PAL ≥ 1.4) were grouped as “Physical Activity Group (PAG)”, while those with a PAL value < 1.4 were categorized as “Sedentary Group (SG)”.

#### 2.3.3. Posturography

Postural control was assessed through posturography [19,20]. Balance was evaluated using dynamic posturography with the Equi Test (Equi Test: Neurocom International, Clackamas, OR, USA). The Sensory Organization Test (SOT) was conducted to measure postural stability under various sensory conditions. The Equi Test system consists of a force platform and a visual environment that can either remain fixed or become mobile, rotating around the ankle joints in response to the individual’s postural adjustments. The system, combined with open or closed eyes, provides information regarding somatosensory, visual, and vestibular contributions to balance.

The test included six conditions: (a) open eyes, fixed visual environment, and fixed support; (b) closed eyes, fixed support; (c) mobile visual environment and fixed support; (d) fixed visual environment and mobile support; (e) closed eyes, mobile support; (f) open eyes, mobile visual environment, and mobile support. Three 20-second measurements were taken in each condition [21]. From these conditions, the following values were derived in Table 1. Each condition was tested three times for 20 seconds to obtain an average of the data.

### 2.4. Data Analysis

The statistical analysis of the data was carried out using the Statistical Program for Social Science (SPSS) version 20.0 (SPSS Inc., Chicago, IL, USA). The Shapiro–Wilk test was used to test the normality of the data. Description variables presented normal distribution; therefore, unpaired Student’s *t*-Tests were performed to compare individuals classified as sedentary or non-sedentary, and one-way ANOVA was used to compare individuals with normal weight, overweight, and obesity. For balance variables that did not present normal distribution, the Mann–Whitney U test was used for the same comparison. The significance level was set at *p* < 0.05. Results were presented as mean ± standard deviation.

## 3. Results

### 3.1. Metabolic Equivalent of Task

Participants were categorized into the SG if their metabolic equivalents of task (METs) values were ≤1.4 and into the PAG if their METs values exceeded 1.4 (Table 2). Additionally, the body mass index (BMI) was 30.42 ± 6.35 in the SG and 29.15 ± 11.46 in the Physical Activity Group, with no significant difference observed (*p* = 0.597).

Table 3 shows no statistically significant differences between the SG and the PAG in terms of lower limb strength (*p* > 0.05) or unipodal stability. However, a significant difference was found in average METS values, with the SG showing lower values compared to the PAG (*p* = 0.01), highlighting a disparity in metabolic equivalents between the groups.

### 3.2. Body Composition

The sample was divided according to body composition (Table 4). No significant differences were observed between groups in age (*p* = 0.56) or height (*p* = 0.78). However, significant differences in BMI were found (*p* < 0.001), consistent with the classification of groups by weight status.

Analysis of lower limb strength and unipodal stability across weight classifications (normal weight, overweight, obese) revealed no statistically significant differences in any variable assessed, with *p*-values of 0.341 for open-eye stability, 0.61 for closed-eye stability, and 0.479 for aggregated stability measures (Table 5). These findings suggest comparable performance among groups regardless of weight classification.

The final analysis, which included all study participants, revealed significant positive correlations between lower limb strength and unipodal support stability under open-eye conditions (Table 6). Specifically, average lower limb strength showed a positive correlation with the sum of open-eye single-leg support stability (*p* = 0.025) and average open-eye single-leg support stability (*p* = 0.025). Additionally, maximum lower limb strength was positively correlated with the sum of open-eye single-leg support stability (*p* = 0.04) and average open-eye single-leg support stability (*p* = 0.04).

These findings indicate that higher lower limb strength is associated with improved stability outcomes in single-leg support tests, suggesting that stronger lower limb muscles contribute to better performance in unipodal stability measures. Therefore, a positive correlation between lower limb strength and stability exists in these specific unipodal tests.

## 4. Discussion

This study sought to verify the effects of lower limb strength, level of physical activity, and body composition on balance in older adults. It was observed that (1) higher levels of lower limb strength are related to better balance in unipodal support with eyes open; (2) the level of physical activity and body composition did not significantly influence the difference in lower limb strength and balance between the groups. This research helps to fill some gaps in the literature, such as understanding the relationship between physical activity and body composition and levels of lower limb strength and single-leg balance, especially in this age group. 

Taylor et al. [22] draw attention to studies of this nature, with this population, in the global scenario where life expectancy is growing associated with physiological declines such as reduced mobility and balance, loss of muscle mass, and metabolic and neuromuscular alterations. Therefore, research in this area makes it possible to indicate the right direction for interventions to take to mitigate positive and negative conditions.

With regard to the total sample, 60.94% were classified as physically active and 39.06% as sedentary. The threshold adopted for classification was 1.4 METS, which estimates energy expenditure with physical activity. This showed a significant difference (*p* = 0.01) between the PAG and the SG [23]. A similar study by Aithal et al. [24] found that the prevalence of insufficient levels of physical activity in more than 2,000 older adults was 33.7%. There was a greater likelihood of this in individuals who were pre-obese/obese, of older age, with sensory and motor limitations, which coincides with the findings of the present study. 

In a study with a sample similar to this one of elderly Europeans, 62.2% met the physical activity recommendations [25]. This can be explained by the living conditions that these countries offer to older people, with safe environments, health care, policies, and infrastructures that allow physical activity to be included in the various spheres; leisure, work, exercise, and sport [26].

Activity at this stage of life plays a fundamental role in preventing chronic diseases such as obesity and the risk of falls, which are associated with loss of strength (muscle mass) and balance over the years [27]. In this sense, for the sample in this study, there was no significant difference in average (*p* = 0.30) and maximum (*p* = 0.33) lower limb strength between GA and GS, with values close to each other. In unipodal support stability, including measurements with eyes open (*p* = 0.36) and closed (*p* = 0.21), there were also no statistically significant differences between GA and GS. This is in line with the review conducted by Keating et al. [7], which points in the direction that higher levels of exercise, such as structured physical activity, promote gains in power and muscle strength, helping to improve balance in older adults. Han et al. [28] showed a significant association between sedentary behavior and worse balance and lower limb strength in older adults. These findings could be different from ours due to the minimum difference in METS between groups. Motalebi et al. [29] evaluated the effect of resistance training on balance and lower limb strength between two groups of elderly people, and the strength scores were higher in the more physically active group, but in agreement with our findings, there was no significant change in the balance after unipodal support tests with eyes open and closed. This can be explained in both studies by the homogeneous characteristics of the samples in general.

Regarding body composition, our sample was divided into three groups: normal weight, overweight, and obese, based on Body Mass Index—BMI [30]. Of these, 12.50% belonged to the normal weight, 43.75% to the overweight, and 43.75% to the obese. This is in line with global trends of overweight and obesity in adults and the elderly, a global health problem whose sedentary lifestyle is an important precursor and also acts as a risk factor for various chronic diseases and functional movement limitations [31,32]. 

There were no statistically significant differences in the comparison between the groups of unipodal stability with eyes open (*p* = 0.34) and closed (*p* = 0.61) and the average (*p* = 0.09) and maximum (*p* = 0.06) strength levels of the lower limbs. There were also no significant differences in the aggregate measures of the sum of all the unipodal supports measured by group (*p* = 0.47) and in the average stability in general unipodal support (*p* = 0.47), indicating adjusted scores between the groups, regardless of weight classification. 

These results diverge from several authors who postulate that a sedentary lifestyle and higher percentages of fat and body mass can compromise balance and postural control due to the changes caused in the body’s center of mass, in the propagation of multidirectional forces, especially in the lower limbs, in the gait pattern, which increases the risk of falls, especially with advancing age, accompanied by the progressive loss of muscle strength [18,33]. 

One possible explanation could be the fact that even though most of the sample was overweight or obese, the majority were physically active, and physical activity has the ability to maintain or improve parameters involved in balance and postural control, such as muscle strength [7]. Castillo-Rodríguez et al. [34] argue that although excess weight can impair the body’s functionality, the strength of obese and normal-weight people may be equivalent because obese people tend to have more lean mass in comparative values, counteracting the negative impact of excess weight on tasks involving balance and posture control.

Other authors have shown similar results and conclusions to our findings when investigating the relationship between physical activity levels and postural balance in overweight and obese adults, in the sense that having a higher level of physical activity (being physically active) is more important for predicting good functional capacities and postural stability than body composition [35]. Onofrei and Amaricai [1] pointed out that the level of physical activity influences balance in adults and has a positive interaction with vision in improving postural control. Brach et al. [36] argue that physical activity is of equal or greater importance than a person’s body weight in predicting good physical function, such as balance.

However, when correlating the variables of lower limb strength and unipodal support stability, a statistically significant difference (*p* = 0.02) was found between mean lower limb strength and mean unipodal support stability with eyes open. The same did not occur in the eyes-closed condition. This indicates that higher lower limb strength scores are associated with better stability results in unipodal support with eyes open. 

People with greater strength were able to use somatosensory data from visual and proprioceptive systems more efficiently. The visual system is the most used for maintaining balance, as it generates continuous spatial information and feedback in integration with the central nervous system via afferent and efferent sensory pathways, which orient the body in the environment and in opposition to disturbances to postural stability [12].

In the study conducted by Onofrei and Amaricai [1], visual input had a significant influence on postural balance in adults, when comparing tests with eyes closed and eyes open. So even though greater lower limb strength has a positive impact on balance because they are the base of support for the body (receiving the reactive force of the ground), without the use of vision (eyes closed), this greater level of strength is not able to compensate for the damage caused to balance by excess weight and obesity [12,14].

De Oliveira et al. [4] in their results agree with our findings by proving that the degree of strength and muscle contraction performed by the muscles of the lower limbs (e.g., plantar flexors, soleus, gastrocnemius) play essential roles in maintaining posture and integrating rapid responses to balance disturbances. A study of older adults showed that the ability to generate lower limb strength is directly related to balance [34]. In addition, Adams et al. [37] consider that physical activity for older adults is very important, as it increases muscle strength and postural control, combating the functional decline in balance linked to age.

This study has some limitations, such as the absence of a control group to serve as a baseline for comparisons, the lack of a comparison by gender between the variables, and a group with high levels of physical activity that would allow analysis by intensity. A comparison of balance scores considering the dominant and non-dominant leg could have been interesting, which other researchers should consider. The cross-sectional nature of the research makes it possible to infer associations, but in order to have a clearer impact, a longitudinal design would be essential. As the sample was made up exclusively of older Spanish adults, the generalization of the results to other regions, with their intrinsic characteristics, is compromised. Future studies should consider longitudinal studies that follow this population for longer periods, making it possible to analyze how balance is affected by fluctuations in physical activity scores, body composition, and lower limb strength.

The findings of this research have important practical implications for professionals in various areas of health (e.g., medicine, physiotherapy, physical education) and public and private organizations, broadening the understanding of the relationship between balance and physical activity levels, body composition, and lower limb strength; guiding programs and treatments that emphasize the adoption of a more physically active lifestyle, seeking to maintain and increase adequate muscle mass to help control a healthy body mass index and fat percentage in order to provide better balance and postural control; over the years, they can suffer negative effects as a result of low levels of physical activity and excess fat in the body composition. In addition, this section of the population is constantly involved in work, domestic, and sporting activities (during leisure time), which require good balance. Therefore, maintaining and improving postural balance is essential for healthy aging and a good quality of life.

## 5. Conclusions

This study found that there was a significantly positive correlation between higher levels of lower limb strength and better levels of balance in unipodal support with eyes open. This shows that the muscle strength component is fundamental to improving somatosensory and proprioceptive responses in human postural balance. No significant differences were found in lower limb strength and postural balance between the groups classified by level of physical activity (active and sedentary) and body composition (normal weight, overweight, and obese). This indicates that, for this sample, body composition and level of physical activity had no impact on these variables.

In addition, more research is needed with larger samples and longitudinal follow-ups in other regions around the world to explore this relationship between physical activity and postural balance in older adults, contributing to a more detailed understanding of the relationship between these elements and more assertive interventions and public policies for the quality of life of these people; thus, promoting a science that is increasingly at the service of society.

## Figures and Tables

**Table 1:** Postural control test.

Measured Index	Description
SOT-SOM (Somatosensory Ratio)	The subject’s ability to use somatosensory stimuli to maintain balance
SOT-VIS (Visual Ratio)	The subject’s ability to use visual stimuli to maintain balance
SOT-VEST (Vestibular Ratio)	Ability of the subject to use vestibular stimuli to maintain balance

**Table 2:** Sociodemographic variables according to metabolic equivalent.

	Sedentary	Physical Activity (*n* = 39)	*p*-Value
Age	57.4 ± 13.626	56.28 ± 6.108	0.328
Weight (kg)	92.36 ± 18.12	90.64 ± 21.52	0.739
Height (m)	174.24 ± 6.23	176.33 ± 27.63	0.688
BMI ^1^ (kg/m²)	30.42 ± 6.35	29.15 ± 11.46	0.597

^1^ Body Mass Index. Data are presented as mean (SD).

**Table 3:** Posturography results according to the level of physical activity.

	Sedentary (*n* = 25)	Physical Activity (*n* = 39)	*p*-Value
Average Lower Limb Strength	141.67 ± 27.33	138.17 ± 25.55	0.30
Maximum Lower Limb Strength	151 ± 28.37	148.01 ± 26.26	0.33
Sum of Open-Eye Single-Leg Support Stability	1.85 ± 2.38	1.69 ± 1.22	0.367
Sum of Closed-Eye Single-Leg Support Stability	13.81 ± 5.57	12.53 ± 6.56	0.213
Average Open-Eye Single-Leg Support Stability	0.92 ± 1.194	0.84 ± 0.61	0.367
Average Closed-Eye Single-Leg Support Stability	6.906 ± 2.78	6.26 ± 3.28	0.213
Sum of Single-Leg Support Stability	15.66 ± 6.58	14.23 ± 7.22	0.214
Average Single-Leg Support Stability	3.91 ± 1.64	3.55 ± 1.80	0.214
Average METS	1.31 ± 0.09	1.65 ± 0.149	0.01

Data are presented as mean (SD).

**Table 4:** Sociodemographic variables according to body composition.

	Normal Weight (*n* = 8)	Overweight (*n* = 28)	Obese (*n* = 28)	*p*-Value
Age	53.88 ± 6.17	54.86 ± 4.66	59.25 ± 13.23	0.56
Weight	70.26 ± 7.38	85.82 ± 8.44	103.68 ± 15.61	<0.001
Height	175.84 ± 5.72	174.23 ± 6.83	176.27 ± 7.13	0.78
BMI ^1^	22.72 ± 2.81	28.27 ± 3.56	33.37 ± 5.71	<0.001

^1^ Body Mass Index. Data are presented as mean (SD).

**Table 5:** Posturography results according to body composition.

	Normal Weight (*n* = 8)	Overweight (*n* = 28)	Obese (*n* = 28)	*p*-Value
Average Lower Limb Strength	131.5 ± 27.39	142.96 ± 27.76	138.41 ± 24.33	0.09
Maximum Lower Limb Strength	143.87 ± 31.57	152.12 ± 28.05	147.75 ± 25.03	0.06
Sum of Open-Eye Single-Leg Support Stability	1.85 ± 1.40	2.07 ± 2.38	1.38 ± 0.75	0.341
Sum of Closed-Eye Single-Leg Support Stability	14.325 ± 6.46	13.47 ± 5.69	12.18 ± 6.70	0.61
Average Open-Eye Single-Leg Support Stability	0.92 ± 0.70	1.03 ± 1.19	0.69 ± 0.37	0.341
Average Closed-Eye Single-Leg Support Stability	7.16 ± 3.23	6.73 ± 2.84	6.09 ± 3.35	0.61
Sum of Single-Leg Support Stability	16.18 ± 7.12	15.55 ± 7.047	13.56 ± 6.89	0.479
Average Single-Leg Support Stability	4.04 ± 1.78	3.88 ± 1.76	3.39 ± 1.72	0.479

Data are presented as mean (SD).

**Table 6:** Correlations between lower and maximum limb strength.

	Average Lower Limb Strength	Maximum Lower Limb Strength
Sum of Open-Eye Single-Leg Support Stability	0.025	0.04
Sum of Closed-Eye Single-Leg Support Stability	0.572	0.337
Average Open-Eye Single-Leg Support Stability	0.025	0.04
Average Closed-Eye Single-Leg Support Stability	0.572	0.337
Sum of Single-Leg Support Stability	0.289	0.171
Average Single-Leg Support Stability	0.289	0.171

## Data Availability

The data presented in this study are available on request from the first author. Data are unavailable due to privacy or ethical restrictions.
